# The Yellow Knight Fights Back: Toxicological, Epidemiological, and Survey Studies Defend Edibility of *Tricholoma equestre*

**DOI:** 10.3390/toxins10110468

**Published:** 2018-11-13

**Authors:** Piotr Klimaszyk, Piotr Rzymski

**Affiliations:** 1 Institute of Environmental Biology, Adam Mickiewicz University, 61-614 Poznan, Poland; pklim@amu.edu.pl; 2 Department of Environmental Medicine, Poznan University of Medical Sciences, 60-806 Poznan, Poland

**Keywords:** *Tricholoma equestre*, toxicity, mushroom poisoning, mushroom edibility, food safety

## Abstract

Rhabdomyolysis, a condition associated with the consumption of Yellow Knight mushrooms (*Tricholoma equestre*), was first reported in 2001. In response, some countries began to consider the mushroom as poisonous, whereas in others it is still consumed. In the present study, a nationwide survey of Polish mushroom foragers (*n* = 1545) was conducted to estimate the frequency of *T. equestre* consumption. The epidemiological database on mushroom poisonings in Poland was analyzed from the year 2008. Hematological and biochemical parameters were followed for a week in 10 volunteers consuming 300 g of molecularly identified *T. equestre*. More than half the foragers had consumed *T. equestre* at least once in their lifetime and a quarter had consumed it consecutively. The frequency of adverse events was low and no rhabdomyolysis was reported. The toxicological database indicated that mushrooms from the *Tricholoma* genus caused poisonings less frequently than mushrooms with well-established edibility and not a single case of rhabdomyolysis has been reported within the last decade. The volunteers consuming *T. equestre* revealed no hematological or biochemical alterations and no adverse effects were observed. The findings of this study support the view that *T. equestre* is edible if consumed in rational amounts by healthy subjects.

## 1. Introduction

The Yellow Knight mushroom, *Tricholoma equestre*, has been considered edible since medieval times, collected from the wild, and highly appreciated for its taste [1]. However, its reputation was unexpectedly tainted in 2001 after Bedry et al. published their highly influential paper entitled “*Wild-mushroom intoxication as a cause of rhabdomyolysis*”, which reported twelve poisoning cases, three of which had a fatal outcome believed to be associated with consumption of this mushroom [2]. Observed clinical symptoms include fatigue, nausea, and muscle weakness followed by myalgia (muscle pain). All patients had significantly increased aspartate (AST) and alanine (ALT) aminotransferases and most importantly creatine kinase (CK), a marker of rhabdomyolysis. Rhabdomyolysis is a syndrome of skeletal muscle cell damage that results in release of intracellular contents into the systemic circulation. Involvement of *T. equestre* in the observed effects was further supported by in vivo experiments that involved feeding mice for three days with mushroom powder or extract. All animals revealed an increased plasma CK concentration and disorganization of muscle fibers [2]. The paper by Bedry et al. [2] was highly publicized and persuaded a number of countries (e.g., France, Spain, Italy) to officially condemn *T. equestre* as poisonous and release warnings to avoid its consumption [1,3]. The already severely damaged reputation of this species has been further tarnished by poisonings documented in Poland [4], Lithuania [5], and Switzerland [6], and observations from another set of in vivo studies in mice [7,8]. Nevertheless, in some European countries, North America, and some parts of Asia *T. equestre* is still considered edible [1].

As recently reviewed, not a single reported case of poisoning provides unequivocal evidence that *T. equestre* was a causative agent. Its consumption was mostly evidenced by information self-reported by patients and the mistaken consumption of some morphologically similar species from the *Tricholoma* or other genera has never been ruled out, nor have any spore identification or molecular data been provided [1]. This is surprising given the fact that most mushroom poisonings arise from mistaken consumption of a poisonous species, e.g., *Amanita phalloides* and *A. virosa*, which contain amatoxin peptides and are often mistaken for *Macrolepiota procera* or species from the *Agaricus* genus [9]. The first cases of human poisoning with *T. equestre* were documented over 15 years ago but as yet no causative toxin has been identified and isolated. Instead, such compounds were characterized in *Russula subnigricans* and *Tricholoma terreum* [10,11]. The reliability of *T. equestre* toxicity data derived from in vivo mice experiments can also be questioned as animals were exposed to doses at which consumption in humans is virtually impossible [2,7,8]. Moreover, similar toxic effects have been observed in rodents after being fed mushrooms with well-established edibility (e.g., *Boletus edulis*), thus implying that such responses may not be specific to *T. equestre* [7].

Therefore, the question remains as to whether Yellow Knight is of a noble or vicious nature. The present study was conducted in an attempt to resolve this question. As Slavic countries still cultivate a tradition of collecting and consuming mushrooms from the wild, the Polish population is considered perfectly suited for such an investigation [12]. The aim was to conduct a nation-wide survey of consumption of this mushroom, analyze epidemiological data on mushroom poisonings from the last decade, and follow hematological and biochemical parameters in individuals consuming molecularly-identified *T. equestre*. The findings support the notion that consumption of *T. equestre* is safe for healthy subjects and that there is no firm evidence to consider this species inedible.

## 2. Results and Discussion

### 2.1. Survey

Foraging for wild mushrooms has a long tradition in Poland. According to a poll made by the Polish Public Opinion Research Center (TNS OBOP), over 40% of adult Poles consider collection of wild mushrooms as a favorite way to spend their spare time [13]. It can be estimated that in Poland alone several million people collect wild mushrooms annually. The present study surveyed a total of 1545 mushroom foragers; among them 35.6% (*n* = 550) had heard claims that *T. equestre* is a toxic species. Nevertheless, the majority (57.5%, *n* = 889) admitted to collecting and consuming it at least once per year. Extrapolation of these findings through results of the TNS OBOP (1994) survey [13] indicate that at least several hundred thousand mushroom foragers in Poland consume *T. equestre* annually. The most commonly indicated forms of consumption were fried (59.0%, *n* = 525), pickled (47.7%, *n* = 424), or boiled (24.2%, *n* = 215) fruiting bodies, as well as in soup (30.9%, *n* = 275). The mean reported dose of a single consumption was 148 ± 82 g (2.1 ± 1.1 g/kg body weight in the case of a 70 kg adult). A reasonable percentage (25.3%, *n* = 225) admitted to consecutive consumption of *T. equestre* at least once in their lifetime for a median of three consecutive days (range 2–14 days) at a mean daily dose of 195 ± 99 g (maximum daily dose of 750 g). Adverse effects following any *T. equestre* consumption were reported only by 0.78% (*n* = 7). In all cases only mild gastrointestinal symptoms (diarrhea and abdominal pain) were observed, excluding one case where a person was admitted to a toxicological unit and had a routine gastric lavage performed. These adverse effects were reported after a single consumption of 100–300 g of mushrooms or, in one case, after 300 g was eaten over three consecutive days. No effects related to muscle pain or weakness that could potentially indicate rhabdomyolysis were reported in any of the cases.

### 2.2. Toxicological Database

According to registry data maintained by the State Sanitary Inspection in Poland, mushrooms belonging to the *Tricholoma* genus were responsible for five cases of poisoning within the last ten years, all of which were characterized by mild gastrointestinal symptoms (nausea, vomiting, and abdominal pain). Information on the causative agent was based on the patients report and species identification based on spores was not conducted in any case. During the same period, 15 poisoning cases with mushrooms considered edible were noted. A summary on mushroom poisoning in Poland indicated that species such as *Macrolepiota procera* or *Imleria badia* are a more common cause of gastrointestinal effects than *T. equestre* [14]. If one considers the estimated number of Poles consuming *T. equestre*, the frequency of adverse effects remains very low and there are no cases involving previously reported rhabdomyolysis. These observations also provide a strong indication that *T. equestre* is generally edible and safe for consumption.

### 2.3. Experimental Study

Molecular analysis of collected fruiting bodies confirmed that the sequences obtained were 100% identical to *T. equestre* ITS sequences deposited in the GenBank and Unified system for DNA based fungal species (UNITE Community). This toxicological study was therefore the first in which investigated *T. equestre* have been indiscriminately taxonomically classified—a condition recently made obligatory for any investigations conducted on this species [1]. It should be highlighted that according to Polish law established and enforced by the Ministry of Health, *T. equestre* is considered an edible mushroom species where collection from the wild is allowed with no restrictions [15]. The mean content of metals in collected fruiting bodies of *T. equestre* was significantly below values found in other studies (as reviewed in Reference [1]) and was well below the maximum allowance set for mushroom foodstuffs by the European Union [16] (Table 1). Some metals are known to induce rhabdomyolysis but accumulation of these elements in *T. equestre* fruiting bodies is too low to reasonably consider them as causative agents of toxic effects in humans [1].

**Figure 1:**
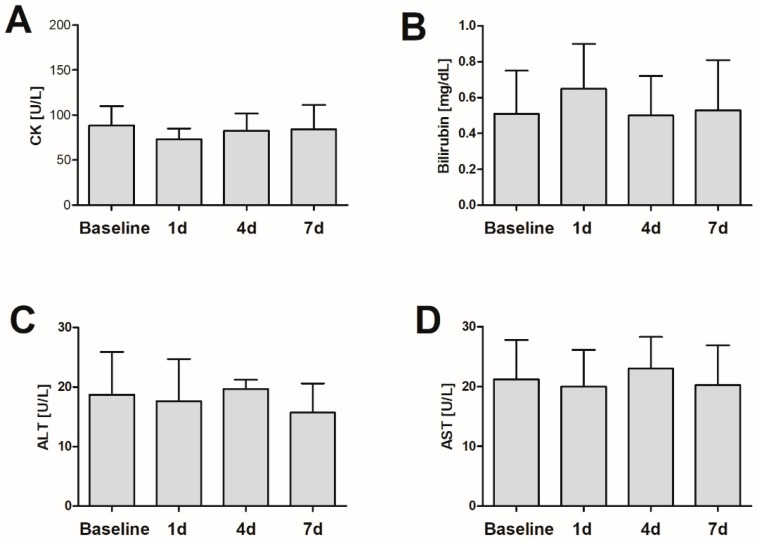
Changes in plasma concentration of creatine kinase (**A**), bilirubin (**B**), alanine aminotransferase (**C**), and aspartate aminotransferase (**D**) in healthy subjects consuming 300 g of fresh *Tricholoma equestre* fruiting bodies.

All studied individuals consumed mushrooms in the most common fried form as a single serving of 300 g of fresh fruiting bodies, a two-fold higher than an average serving as declared by surveyed mushroom foragers. After consumption of 300 g of mushrooms no significant change in any hematological or biochemical parameter was observed during the study period (Table 2; Figure 1). No relevant adverse events were noted by volunteers (two individuals reported a headache the day following consumption). No gastrointestinal irritation was reported. No studied individual experienced gastrointestinal irritation, the most frequently reported idiosyncratic toxic effect following consumption of edible mushrooms [17].

This is the first study on *T. equestre* toxicity where investigated specimens have been identified using molecular tools. This approach is undeniably essential for correct taxonomic identification of species [18]. One should note that in most poisoning cases *T. equestre* was believed to be a causative factor only on the basis of reports made by a patient and/or a morphologically-based taxonomical determination of mushroom remains by medical staff. The reliability of such information is very low. As reported in Switzerland, 37% of mushroom harvests contained inedible species and 12% contained poisonous species [6]. The possibility of mistaking other species within the *Tricholoma* genus is highly possible because some of them share both morphological features and distribution with *T. equestre* (e.g., *T. frondosae*, *T. sulphureum*, *T. sejunctum*, *T. aestuans*, *T. terrerum,* and others). There is also the possibility of mistaking them for other gilled mushroom species with similar coloration and morphology from the genus *Russula* or *Amanita* [1,18]. Findings of the present study evidence that incorrect identification of *T. equestre* is plausible and that adverse effects following its consumption are rare and of a mild gastrointestinal character, similar to those often caused by other edible species. Health-threatening mushroom poisonings such as those by *A. phalloides* can result from mistaken species identification, often a failure to distinguish a difference between *M. procera* or *Agaricus* sp. yet no one has ever claimed that these are poisonous.

The reported poisonings involving *T. equestre* described an onset of rhabdomyolysis following consecutive ingestion of 100–400 g of fresh mushrooms daily, indicating an accumulative effect. Thus, if *T. equestre* was the sole causative agent of induced toxicity one would expect studied volunteers to reveal, at least to some extent, an increase in biochemical parameters, particularly CK concentration—a known myotoxicity marker. However, the present study highlights that correctly identified *T. equestre* does not appear to pose any threat to healthy subjects if consumed in rational amounts.

Although the first poisonings from *T. equestre* were reported over 15 years ago, toxic compounds, including potential causative agents of rhabdomyolysis, have never been identified. Rhabdomyolysis has also been reported in humans following consumption of white button mushroom species *Agaricus bisporus* [19] or species from the *Boletus* and *Leccinum* genera [20]. Moreover, the in vivo model applied to confirm *T. equestre* toxicity does not provide convincing evidence as significantly increased plasma CK concentrations have also been noted in mice fed with high doses of edible mushrooms including *Boletus edulis*, *Lentinula edodes*, *Cantharellus cibarius*, *Albatrellus ovinus*, *Leccinium versipelle*, *Imleria badia,* and *Flammulina velutipes* [7,8,21,22]. Yet their edibility and general safety has never been questioned and no warnings have ever been released. Instead it has been suggested that individual sensitivity could play a role in the development of such symptoms and that rhabdomyolysis may represent an unspecified reaction, unrelated to any specific mushroom species [1].

However, this research supports the notion that *T. equestre* eaten by healthy subjects in rational amounts are non-toxic per se, acknowledging the study limitations. Firstly, the number of enrolled volunteers was small and due to ethical reasons could only include healthy individuals. Therefore, the existence of idiosyncratic reactions to this mushroom cannot be ruled out. Moreover, this study evaluated the effect of consuming a single meal of 300 g while poisoning cases often included subjects that consumed mushrooms consecutively. Poisoned individuals revealed extremely high CK levels so one could hypothesize that a single dose could also lead to a distinctively slight but detectable increase. However, one-quarter of surveyed mushroom foragers declared eating *T. equestre* consecutively with no side-effects. The present study cannot fully rule out the possibility of accumulative effects resulting from repeated consumption of unreasonable amounts of *T. equestre* and such consumption should therefore be avoided. Finally, the present study only evaluated the effect of consuming *T. equestre* fruiting bodies fried in butter, which according to survey data was the most popular form of consumption for this mushroom in Poland. However, the exact form under which mushrooms in reported cases of poisonings were consumed, were not reported and remain unknown [2,5]. Various methods of mushroom preparation (frying, boiling, pickling, etc.) may exert different effects on chemical properties of the consumed product [23]. In our study, volunteers consumed mushrooms fried in butter, therefore we cannot rule out the possibility that in some other form (or if undercooked) a *T. equestre* meal may exert some toxic effects. One should note that results of the survey study indicate no association between any form of mushroom preparation and toxicity. As recently suggested [1], future descriptions of clinical poisoning cases where *T. equestre* is suspected as a causative factor should report as many details as possible, including the form in which the mushrooms were consumed and if possible molecular confirmation (e.g., on concentrated gastric content or uneaten fruiting bodies) that *T. equestre* was consumed by the poisoned subject.

## 3. Conclusions

The present study did not found evidence of *T. equestre* toxicity. It is possible that previously reported cases of poisonings were caused by: Consumption of other poisonous mushroom species that are morphologically similar to *T. equestre*; are associated with individual susceptibility whose basis is yet to be explained; or that the observed effects represent an unspecified reaction unrelated to a specific mushroom species, that may onset at high and repeated consumption. Considering this, caution in collection and consumption of *T. equestre* (particularly in large quantities and consumed consecutively) should still be advised. Similar to this study, future toxicological research should first establish the phylogenetic position of investigated specimens using molecular tools.

## 4. Materials and Methods

### 4.1. Survey

The frequency of adverse effects following *T. equestre* consumption was estimated using a three-step approach:

(1) Data on mushroom poisonings within the past 10 years was obtained from the registry of mushroom poisonings maintained by the State Sanitary Inspection in Poland, collected annually from clinical units in Poland. Data from this registry were used to estimate the number of registered poisonings associated with *Tricholoma* and to specify the adverse effects associated with its consumption. The frequency of reported poisonings was compared to that registered for other species classified as edible in Poland.
(2) Thirteen toxicological units in Poland were contacted to obtain information on *T. equestre* poisonings within the past 10 years. This ensured that cases not reported to the registry of mushroom poisonings maintained by the State Sanitary Inspection in Poland would be included in this study.
(3) Mushroom foragers in Poland were surveyed in 2018 using an online self-designed and structured, short, pre-tested (debriefing interview, *n* = 10) questionnaire. It assessed: (i) Number of mushroom foragers collecting and consuming *T. equestre* in Poland (two dichotomous yes/no questions); (ii) the most common forms of consumption (multiple choice question); (iii) a mean dose of a single *T. equestre* consumption as grams of fresh weight (open-ended question); (iv) frequency of consecutive consumption of *T. equestre* (dichotomous yes/no question), number of days of consecutive consumption and a mean daily dose in grams of fresh weight (open-ended questions); (v) occurrence of adverse events (dichotomous yes/no question), specification of these events (open-ended question), whether medical help was required (dichotomous yes/no question) and of what kind (open-ended question). Invitations to complete the questionnaire were sent to different amateur mycological groups, employees of National Parks in Poland and National Forest Holding–State Forests in Poland (as these two occupational groups were likely to have a high rate of wild mushroom consumption) and were made available via academia and public media. The invitation contained information that the survey was addressed to all mushroom foragers, not exclusively consumers of *T. equestre*.

### 4.2. Experimental Study

Participants of the study were recruited in 2017 at the Department of Environmental Medicine of Poznan University of Medical Sciences by advertisement. Ten healthy adult volunteers (5 males and 5 females, 27–78 years old, body mass 73 ± 24 kg, BMI 24.6 ± 4 kg/m^2^) willing to consume *T. equestre* were enrolled in the study. All subjects were collecting *T. equestre* mushrooms on their own and agreed to undertake additional biochemical tests following their next consumption. According to Polish law regulation established and enforced by the Ministry of Health, *T. equestre* is considered an edible species and collection from the wild is allowed with no restrictions [14]. Study protocol was approved by the Bioethical Committee at Poznan University of Medical Sciences (Approval No. 1053/17; Date of approval: 9 November 2017). Every recruited subject undersigned written informed consent. The inclusion criteria included: No consumption of mushrooms within the preceding 7 days; no chronic disease; no evidence of any active disease; hematological parameters, plasma ALT, AST, CK, and bilirubin within reference ranges. The exclusion criteria included: Chronic renal, gastrointestinal, immune, endocrine (except previously treated and stable hypothyroidism in the euthyreosis stage), genetic, musculoskeletal, metabolic, neural, psychiatric, parasitic, pulmonary, or cardiological disease; any history of a rhabdomyolysis event; use of specific medications (monoamine oxidase inhibitors, selective serotonin reuptake inhibitors, statins, fibrates, cyclosporine A, diuretics); alcoholism; cigarette smoking; and drug use.

All mushroom specimens used in the experiment were collected in northwestern Poland, in forests (reported as young pine *Pinus sylvestris* stands), and were remote from public roads, human settlements and industrial establishments where no contamination was expected. Collected mushrooms were identified by qualified mycologists and confirmed by ITS1-ITS4 primers [24,25]. Sub-samples of collected fruiting bodies were also subjected to an analysis of metal content (Al, As, Ba, Cd, Co, Cr, Cu, Hg, Mn, Ni, Pb, V, and Zn) in a certified laboratory (ILAC, PCA AB700) using Inductively Coupled Plasma-Mass Spectrometry or Cold Vapor Atomic Absorption Spectrometry (in the case of Hg).

Between 9:00–10:00 a.m. and in the presence of a researcher each participant consumed a standard serving of 300 g of fresh *T. equestre* fried for 10 min with butter. Individual mushroom dose varied from 2.4 to 5.4 g/kg body mass (mean 4.0 g/kg body mass). The serving size was determined as twice an average single consumption dose of *T. equestre,* as declared by surveyed mushroom foragers. Mushrooms were fried as this method of consumption was the most frequently reported in the survey. A number of hematological and biochemical parameters (plasma CK, AST, ALT, bilirubin) were evaluated 1, 4, and 7 days after consumption by an accredited laboratory (Bio-Rad EQAS). All participants were instructed to fill a daily questionnaire concerning the occurrence of any adverse events and advised neither to consume alcohol, nor undertake severe physical activities, nor change dietary habits during the study period.

## Figures and Tables

**Table 1:** Elemental composition in *T. equestre* fruiting bodies (mg/kg dry weight) consumed by individuals enrolled in this study.

Element	Mean ± SD	Range
**Al**	40.75 ± 9.03	29–51
**As**	0.35 ± 0.05	0.29–0.42
**Ba**	1.13 ± 0.22	0.9–1.4
**Cd**	0.85 ± 0.04	0.79–0.88
**Co**	0.16 ± 0.01	0.15–0.18
**Cr**	0.095 ± 0.01	0.082–0.107
**Cu**	35.5 ± 3.32	32–40
**Hg**	0.82 ± 0.05	0.762–0.892
**Mn**	18.7 ± 2.75	16–22
**Ni**	0.808 ± 0.24	0.55–1.1
**Pb**	1.1 ± 0.37	0.6–1.5
**V**	0.026 ± 0.0036	0.021–0.029
**Zn**	187.5 ± 17.08	170–210

**Table 2:** The haematological parameters of studied individuals baseline, 1, 4 and 7 days after consumption of 300 g of *T. equestre*. No significant changes were noted.

Parameter	Baseline	1 Day	4 Days	7 Days
**WBC** [10^9^/L]	6.32 ± 0.76	5.89 ± 0.90	6.25 ± 0.87	6.04 ± 1.06
**RBC** [10^12^/L]	4.82 ± 0.50	4.89 ± 0.57	4.82 ± 0.35	4.81 ± 0.56
**HGB** [g/dL]	14.16 ± 1.3	14.26 ± 1.24	14.41 ± 0.87	14.19 ± 1.29
**HCT** [%]	44.25 ± 3.55	44.69 ± 4.07	44.84 ± 2.34	44.27 ± 4.09
**MCV** [fL]	91.95 ± 3.12	91.76 ± 3.31	93.05 ± 1.99	92.37 ± 3.38
**MCH** [pg]	29.42 ± 1.39	29.29 ± 1.24	29.90 ± 1.16	29.64 ± 1.74
**MCHC** [g/dL]	32.00 ± 1.23	31.93 ± 0.58	32.13 ± 0.87	32.07 ± 1.13
**RDW** [%]	11.99 ± 0.73	11.83 ± 0.50	12.08 ± 0.36	11.79 ± 0.50
**PLT** [10^9^/L]	230.68 ± 40.33	232.87 ± 40.28	218.50 ± 44.40	230.81 ± 36.21
**PCT** [%]	0.19 ± 0.02	0.20 ± 0.02	0.18 ± 0.02	0.18 ± 0.02
**MPV** [fL]	8.29 ± 1.49	8.90 ± 1.97	8.24 ± 1.67	8.18 ± 1.43
**NEU** [%]	49.07 ± 8.59	53.40 ± 9.13	46.76 ± 13.44	50.05 ± 10.99
**NEU** [10^9^/L]	3.12 ± 0.77	3.18 ± 0.92	3.02 ± 1.25	3.11 ± 1.23
**LYMPH** [%]	36.42 ± 5.38	32.67 ± 5.93	36.87 ± 8.79	36.05 ± 8.30
**LYMPH** [10^9^/L]	2.28 ± 0.32	1.90 ± 0.30	2.26 ± 0.33	2.11 ± 0.29
**MON** [%]	10.49 ± 3.62	9.92 ± 3.25	12.11 ± 3.43	9.73 ± 2.76
**MON** [10^9^/L]	0.66 ± 0.21	0.58 ± 0.19	0.74 ± 0.15	0.58 ± 0.14
**EOS** [%]	2.83 ± 1.35	2.82 ± 1.38	2.88 ± 0.98	2.99 ± 1.53
**EOS** [10^9^/L]	0.18 ± 0.10	0.17 ± 0.09	0.18 ± 0.05	0.18 ± 0.09
**BASO** [%]	1.20 ± 0.41	1.19 ± 0.37	1.39 ± 0.47	1.19 ± 0.45
**BASO** [10^9^/L]	0.08 ± 0.03	0.07 ± 0.02	0.08 ± 0.02	0.07 ± 0.02

WBC—white blood cells; RBC—red blood cells; HGB—hemoglobin; HCT—hematocrit; MCV—mean corpuscular volume; MCH—mean corpuscular hemoglobin; MCHC—MCH concentration; RDW—red blood cell distribution width; PLT—platelets; PCT—platelecrit; MPV—mean platelet volume; NEU—neutrophils; LYMPH—lymphocytes; MON—monocytes, EOS—eosinophils; BASO—basophils.
